# Prolonged Use of Postoperative Gabapentin After Discharge From a Skilled Nursing Facility

**DOI:** 10.1111/jgs.70578

**Published:** 2026-07-11

**Authors:** Tasce Bongiovanni, Siqi Gan, Emily Finlayson, W. John Boscardin, Michael A. Steinman, James D. Harrison

**Affiliations:** 1 Department of Surgery, University of California San Francisco School of Medicine, San Francisco, California, USA; 2 Division of Geriatrics, University of California San Francisco School of Medicine, San Francisco, California, USA; 3 Northern California Institute for Research and Education, San Francisco, California, USA; 4 Department of Medicine, University of California San Francisco School of Medicine, San Francisco, California, USA; 5 Department of Epidemiology & Biostatistics, University of California San Francisco School of Medicine, San Francisco, California, USA; 6 San Francisco VA Medical Center, San Francisco, California, USA; 7 Division of Hospital Medicine, University of California San Francisco School of Medicine, San Francisco, California, USA

**Keywords:** gabapentin, multimodal pain control, skilled nursing facility, transitions of care

## Abstract

**Background:**

The number of older adults being discharged to a skilled nursing facility (SNF) after surgery continues to rise. Prior studies have shown that gabapentinoid prescribing after surgery is increasing, though this work only studied patients transitioning from hospital to home. Therefore, we aim to describe both use and prolonged use of gabapentinoids for older adults who are discharged to SNF and then home after surgery.

**Methods:**

We conducted a retrospective analysis by merging patient data from Medicare Carrier, MedPAR, and Outpatient Files with Medicare Part D for 2013–2020. We included patients ≥ 66 years at time of procedure undergoing one of 14 common surgeries performed in older adults whose discharge was to SNF then home.

**Results:**

The total study cohort included 79,417 patients who were discharged to a SNF. Median length of SNF stay was 15 days (IQR 10, 22 days). A total of 3182 (4%) received a new gabapentinoid prescription. Of these, 38% (*n* = 1219) had prolonged use. On multivariable logistic regression, we found that female sex, gabapentinoid days supply, emergency surgery, non-orthopedic and nonvascular surgery, and higher area deprivation index were associated with prolonged use.

**Conclusions:**

Of our cohort, 38% had prolonged use, more than 1.5 times the proportion of prolonged use found in a past study among patients being discharged directly home. Importantly, patients who were sicker and more disadvantaged were more likely to have prolonged use. Our study highlights that additional work needs to focus on understanding and preventing inappropriate prolonged use of gabapentinoids after SNF discharge.

## 1 ∣ Introduction

The number of older adults having surgery in the United States is rapidly increasing. Postoperatively, some of these patients may go to a skilled nursing facility (SNF) for short term rehabilitation for physical and occupational therapy, wound care, or medical management before being discharged home. The proportion of patients being discharged to SNF varies based on type of surgical procedure but ranges from 10% to 40% [1, 2], and in 2023 specifically was 11.2% among NSQIP hospitals [3]. These transitions of care from the hospital to a SNF, then to home after surgery have the potential to be high-risk for medication misuse and overuse [4-7]. This is especially concerning for older adults in the postoperative period as surgical patients are often prescribed high-risk medications for pain management such as opioids and gabapentinoids, potentially inappropriate medications (PIMs) on the Beers criteria list when used together [4, 5, 8, 9]. Medication discrepancies at SNF transition are common. In fact, one study found that medication discrepancies occurred in almost three of four SNF admissions and opioids were part of the group of medications accounting for over 50% of all discrepant medications [6]. Communication between hospital and SNF is a major source of error in these transitions [4, 6, 10, 11]. SNF clinicians report that key medication information is missing, delayed, discrepant or of poor quality [12, 13]. These problems in transitions of care from hospital to SNF may also increase the possibility of confusion and errors upon a patient's discharge to home.

Improving management of pain medication for older adult surgical patients during these care transitions is essential due to the health risks and high costs associated with medication errors. Medication errors, particularly with high-risk medications like opioids and gabapentinoids, are significant drivers of aging-related disability and functional decline among older adults [14-16]. Further, prior studies have shown that gabapentinoid prescribing after surgery is increasing over time [17]. However, this work only studied patients who transitioned from the hospital to home. Importantly, these transitions of care themselves have been identified as increasing the risk that older adults will have prolonged use of pain medications [18], which can lead to adverse events and functional decline [19-28]. Despite these myriad risks of concomitant and prolonged use, little is known about gabapentinoid use in the care transition from hospital to SNF to home. As 1 in 6 people in the United States are > 65 years old, a population that grew nearly five times faster than the total population over the past 100 years, this rapidly growing population is critical to study [29]. Therefore, we aim to describe both the use and prolonged use of gabapentinoids for older adults who are discharged to SNF and then home after surgery.

## 2 ∣ Methods

### 2.1 ∣ Data Source

We conducted a retrospective analysis by merging patient data from Medicare Carrier, MedPAR, and Outpatient Files with Medicare Part D and the Master Beneficiary Summary File base file for 2013–2020, using a 20% Medicare sample. MedPAR, Outpatient, and Carrier files were used to identify included surgical procedures [30]. We used the MedPAR inpatient files to identify hospitalizations for included surgical procedures with hospital discharges within 24 h of SNF admissions. Outpatient and Carrier files include fee-for-service claims submitted by clinicians from free-standing ambulatory surgical centers. Medicare claims allowed us to use validated claims-based methods to identify surgeries [18], characterize patient complexity through a Charlson Comorbidity Index [31], and identify prescriptions before and after the SNF stay. We stopped our analysis at the end of March 2020 due to the COVID pandemic changing or delaying most elective surgeries [32, 33], and because many SNFs changed their admission and care policies during that period. Medicare, a federal health insurance program which covers approximately 96% of all US citizens ≥ 65, provides this dataset as a representative sample of 20% of the total population. To determine cohort composition, we merged Medicare Carrier, MedPAR, and Outpatient files with Part D files and the MBSF base file. Variables we analyzed included age, sex, race and ethnicity, comorbidity score, socioeconomic disadvantage based on zip code [34], discharge location, and prescription fills. We used an existing cohort of patients who had undergone a surgical procedure [18] and were then discharged to a SNF. We tracked both gabapentinoid (gabapentin [gabapentin, neurontin, horizant, and gralise] and pregabalin [pregabalin]) and postoperative opioid prescribing after discharge to SNF and discharge from SNF to home. Our list of opioids is included as a supplement (eTable S1). This article utilizes the Strengthening the Reporting of Observational Studies in Epidemiology (STROBE) reporting guideline for cross-sectional studies (see supplemental documents) [35]. The study was approved by the University of California San Francisco Institutional Review Board.

### 2.2 ∣ Study Population

We included patients ≥ 66 years at time of the procedure undergoing one of the 14 most common non-cataract surgeries performed in older adults [36, 37], (eTable S2) whose discharge from the postoperative hospitalization was to SNF. The 14 surgical procedures selected represent a wide range of anatomic regions, surgical risk and surgical subspecialties and have been commonly used in other studies [17, 18, 36, 38]. These procedures include orthopedic procedures (joints, hips), spinal procedures (laminotomy/laminectomy), vascular procedures, open and laparoscopic general surgery and colorectal procedures, and gynecologic and urologic procedures. We defined inpatient procedures using ICD9-CM or ICD10-PCS codes and outpatient procedures using Current Procedural Terminology (CPT)/Healthcare Common Procedure Coding System (HCPCS) codes (eTable S3). We included specific groups of patients who had two different procedures on the same day if those procedures both fell into our inclusion criteria (specifically total shoulder arthroplasty/total hip arthroplasty and total shoulder arthroplasty/knee arthroplasty) and we combined the spinal procedures (laminectomy and laminotomy) into one category for analysis given their extensive overlap in our dataset. For patients with multiple dates of surgery which met inclusion criteria over the time period, we analyzed only their most recent surgical date. We excluded patients who died within 30 days after hospital discharge, whose discharge disposition was hospice [39], or who had ≥ 3 procedures on the same day.

As described in prior work [18], to ensure we included patients who were using their Part D benefit, we only included patients with continuous Part D coverage for 3 months before and 30 days after the procedure date who had at least one prescription filled in Medicare Part D during the 3 months prior to surgery. To ensure we had only naïve users of gabapentinoids, we excluded patients who had any prior fill of a gabapentinoid within the 3 months prior to surgery (excluding 7 days prior to surgery as that time period has found to be used to prescribe discharge medications) [18]. We included patients ≥ 66 years to allow for 1 year prior to the procedure to compile comorbidities to calculate a Charlson Comorbidity Score [40] using an updated 17-disease version more appropriate for use in administrative databases [41]. We defined race and ethnicity using the Research Triangle Institute race code, which is an algorithm providing an expanded definition of race and ethnicity to the Medicare data [42].

**FIGURE 1 ∣:**
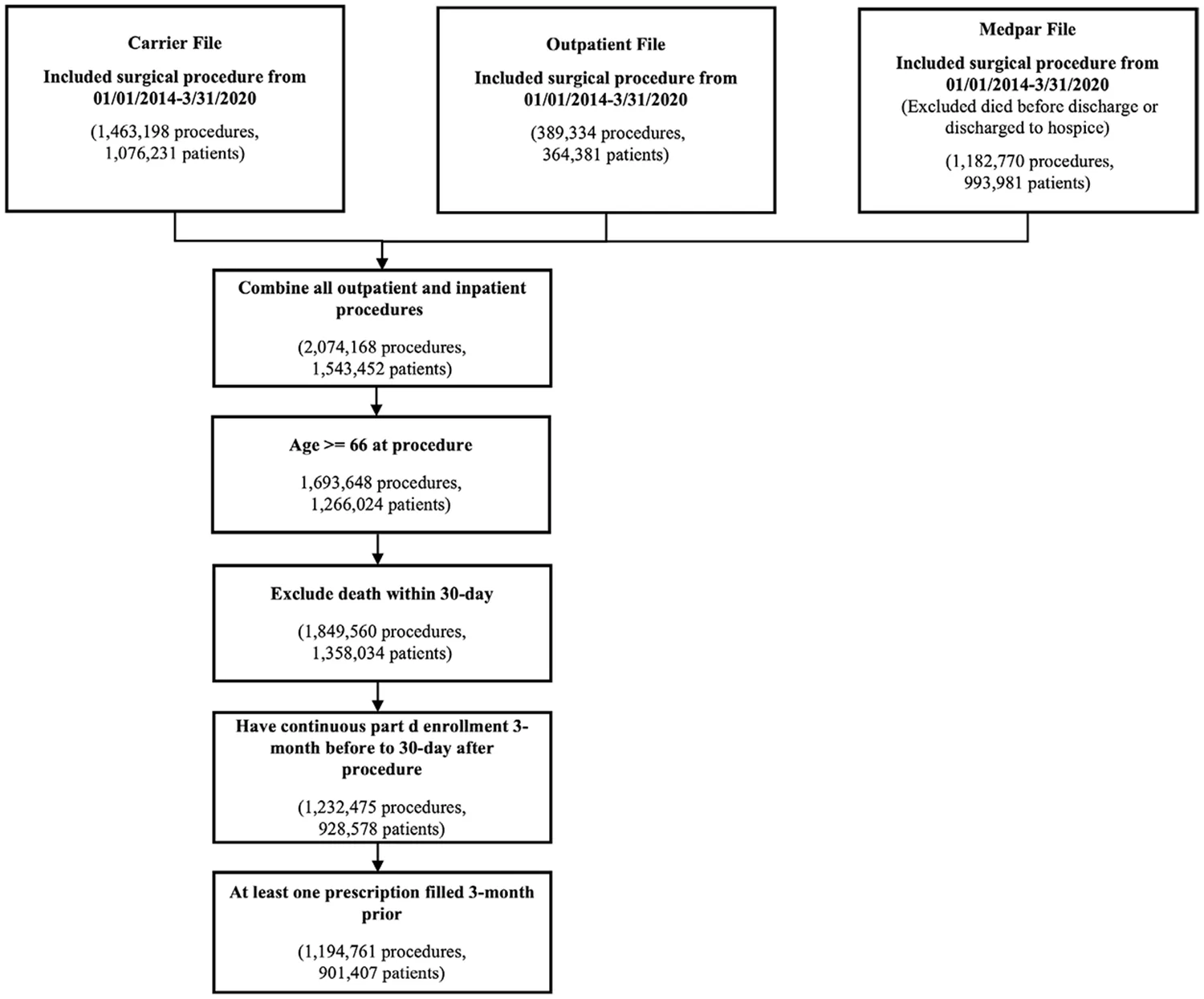
Inclusion criteria flow sheet.

From this cohort (see flow chart, Figure 1), we considered a postoperative prescription as any medication fill 7 days before or after discharge from procedure or SNF as this technique has been used for prescribing completed before discharge to ensure the patient has appropriate medications already at home, as is sometimes done for hospitalized patients [36, 43]. We pulled the NDC codes from Part D claims and then linked them with Medispan crosswalk files to identify generic drug names and prescription information. We excluded patients with a SNF stay longer than 90 days, as their stay overlapped with our definition of prolonged use. We also excluded patients who were discharged from SNF to SNF or from SNF to acute care.

### 2.3 ∣ Outcomes and Data Analyses

The primary outcomes are both the rate of prescribing of gabapentinoids and then the prolonged use of gabapentinoids among patients discharged to a SNF following a surgical procedure. Among patients with a discharge prescription, we defined prolonged use of gabapentinoids as any prescription filled between 90 and 180 days after procedure or SNF discharge. We used the “days supply” variable in Part D to calculate the days' supply and summed all prescribing over this time period. We then defined which procedures more commonly had postoperative gabapentin prescribing. Additionally, we assessed opioid prescribing and oral morphine equivalents of the prescription (e appendix3) using the same methods as the premise of postoperative gabapentinoid use is to decrease the need for opioids. The oral morphine equivalent calculator is used as a tool to compare different opioids using an equianalgesic dose chart to calculate opioid dosage in a consistent and systematic way [44]. Finally, we evaluated concomitant prescribing of opioids in the postoperative period, since concomitant use can increase the risk of adverse drug events.

Chi-squared tests were used for categorical variables and *t*-tests were used for continuous variables. Statistical significance was set to *p* < 0.05. To identify risk factors for prolonged use we constructed logistic regression models, adjusted for procedure characteristics (surgery type including emergent vs. elective), patient characteristics (age, sex, race/ethnicity, Charlson Comorbidity Score) and area deprivation index [34] (based on patient zip code), medication days supply, and concomitant opioid prescribing. We handled competing risks through a descriptive model as the number of deaths within 30 days was too small for a Fine-Gray calculation. Analyses were conducted using SAS 9.4, Stata 17, and plots were generated with R.

## 3 ∣ Results

### 3.1 ∣ Cohort Characteristics

The total study cohort included 79,417 patients who were discharged to a SNF, with a mean age of 77.4 years, 73% female and 86% White. Orthopedic procedures made up the majority of postoperative patients discharging to SNF (88%). Most of the procedures were elective (85%). Median length of stay for the SNF was 15 days (IQR of 10, 22 days). Most patients (78%) went home after discharge from a SNF without any home health services. eTable S4 describes the demographic and clinical characteristics of the study cohort.

### 3.2 ∣ Gabapentinoid Prescriptions

A total of 3182 (4%) received a new gabapentinoid prescription after a postoperative SNF stay. Of those who received a new gabapentinoid prescription, 77% (*n* = 2441) were women and the majority were white (*n* = 2591, 81%). Patients with a new gabapentinoid prescription received a median 30-day supply (IQR 20, 30). White patients were less likely to receive a gabapentinoid prescription (White = 3.8%, Black = 5.5%, Hispanic = 5.1%, all other groups/unknown = 5.4%, *p* < 0.0001). There was significant variation in prescribing by procedure type, with the most common procedure in the new gabapentinoid prescription cohort being a spine procedure (11.6% of the total spine procedures), followed by orthopedic procedures (3.8% of the total orthopedic procedures). Of those with a new gabapentinoid prescription, 60.9% had a concomitant opioid prescription during the same time period; 41.9% of those without a gabapentinoid prescription had an opioid prescription. Finally, average oral morphine equivalents for those discharged with opioid was higher in the gabapentinoid prescribing group (524 vs. 477, *p* = 0.01), though the standard deviation was high (718 vs. 724), important given studies have found that a higher overall quantity of prescribed opioids is associated with higher consumption [45]. (eTable S4).

### 3.3 ∣ Prolonged Use of Gabapentinoids

**FIGURE 2 ∣:**
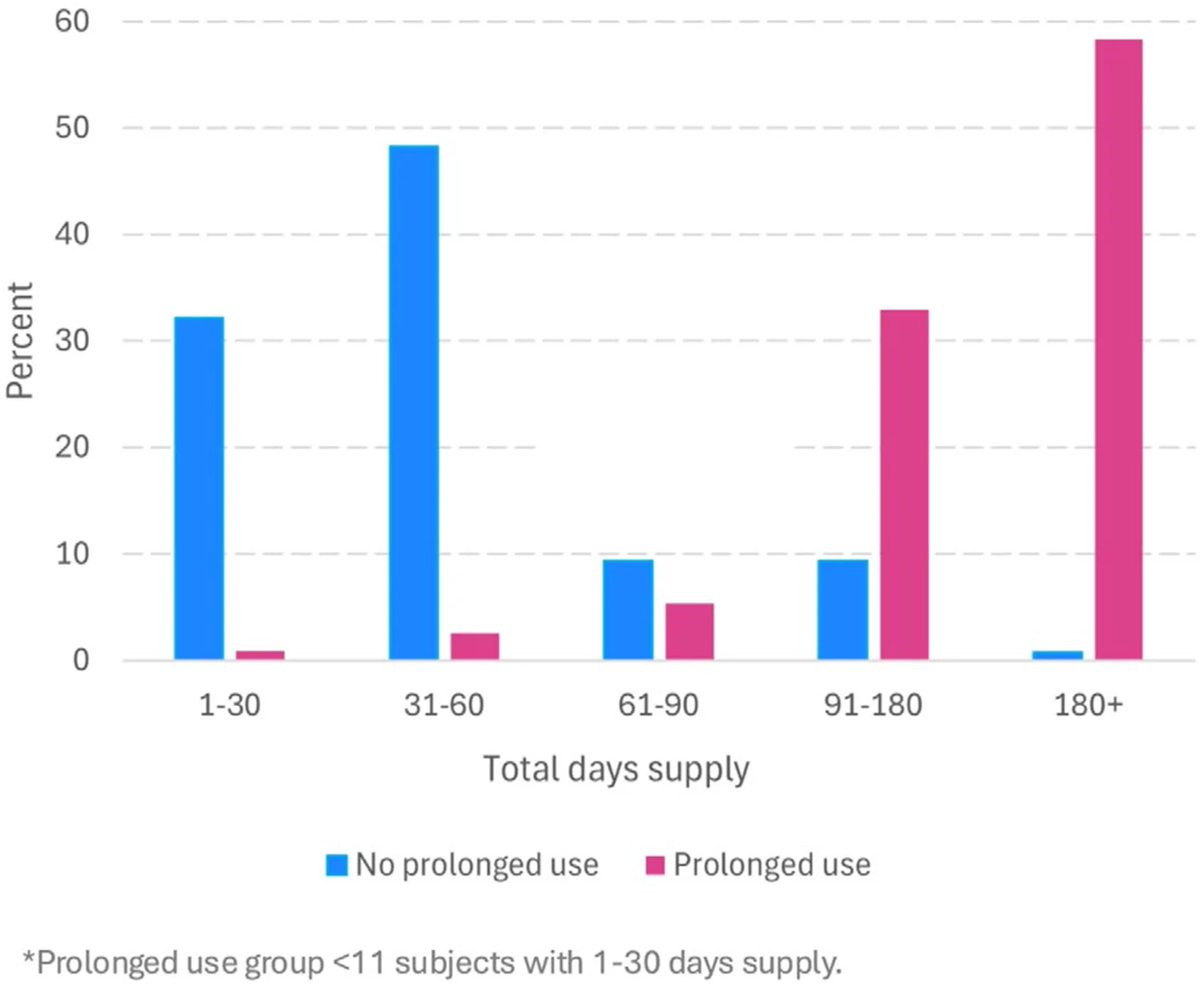
Distribution of prescribing and prolonged use of gabapentin among older surgical patients discharged to SNF then home.

**FIGURE 3 ∣:**
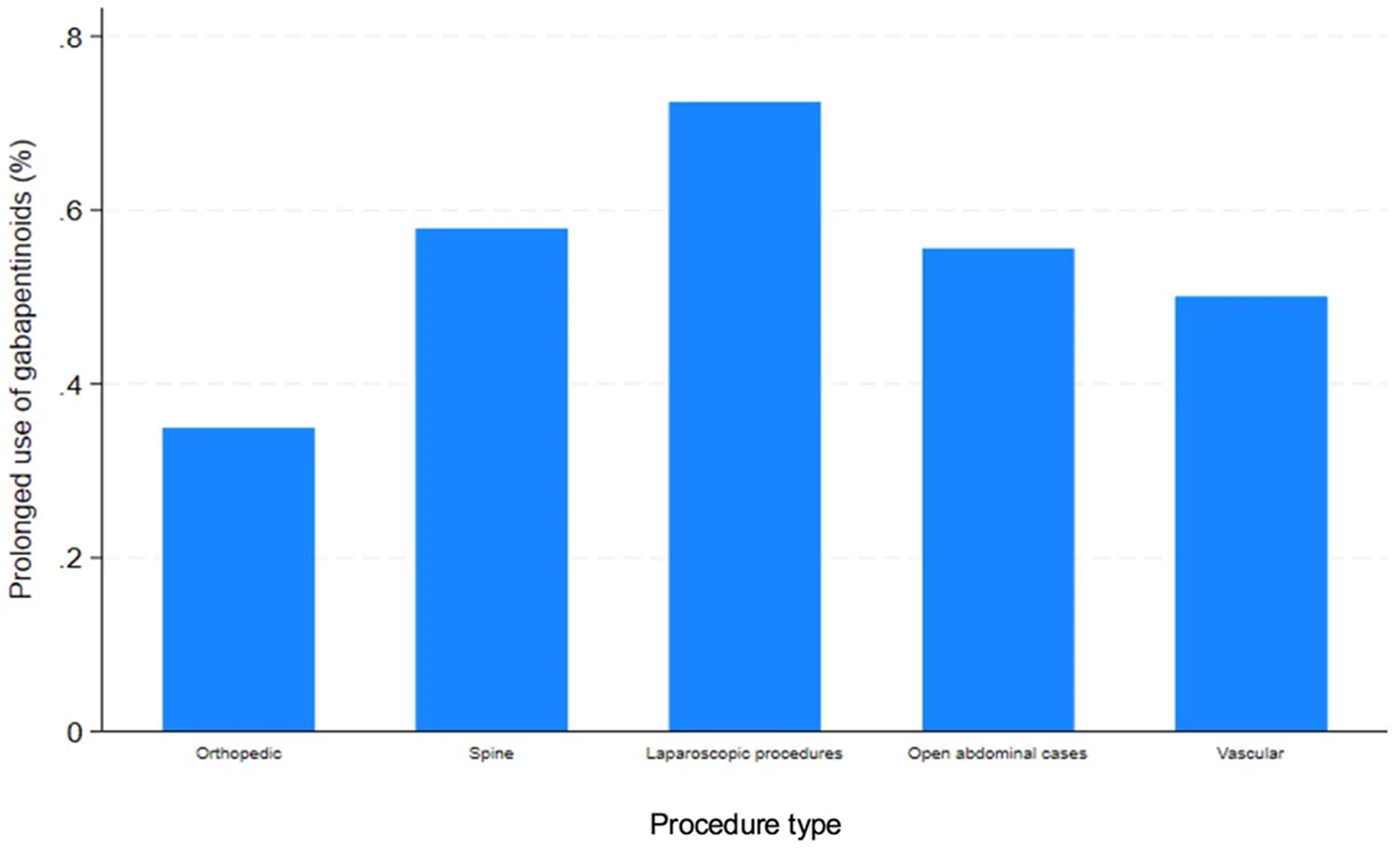
Prolonged use by procedure.

Of the total (*n* = 3182) who received a new gabapentinoid prescription, 38% (*n* = 1219) had prolonged use (Figure 2, Table 1). Of those with prolonged use, the average age was 76 years old (sd 6.5), 77.8% female and 80.8% white, 54.3% had a Charlson score of 3 or greater, compared to 39.8% with a Charlson score of 3 or greater in the non-prolonged use group (*p* < 0.001). While both groups had a median 30-day supply of their discharge prescription, the IQR for the prolonged use cohort was 30,60, while for the non-prolonged use group it was 15,30 (*p* < 0.001). Of those in the prolonged use group, 59.6% had concomitant opioid prescribing at 7 days, and 51.5% still had an opioid prescription after 90 days. The prolonged use group also had a higher opioid days supply of 10 (IQR 6, 20) compared to 7 (IQR 5, 14) days in the non-prolonged use group (*p* < 0.001). The prolonged use group was also more likely to have had an emergent surgery (17.4% vs. 7.9%, *p* < 0.001). Median SNF length of stay was longer in the prolonged use group (20 vs. 15, IQR 13,31 vs. 10,21, *p* < 0.001). Prolonged use by procedure can be found in Figure 3. On multivariable logistic regression (Table 2), we found that female sex, gabapentinoid days supply, emergency surgery, non-orthopedic and nonvascular surgery and higher area deprivation index (greater level of socioeconomic disadvantage) were associated with prolonged use.

## 4 ∣ Discussion

In this study of postoperative prescribing of gabapentinoids after discharge to SNF, we sought to describe prescribing and prolonged use of gabapentinoids after discharge from a SNF stay following surgery by examining nationally representative Medicare data. We found that 4% of older adults in our sample discharged from the hospital to SNF filled a gabapentin prescription around SNF discharge. These patients are more likely to be women, non-White and have had spine or orthopedic procedures. Patients prescribed gabapentinoids were also more likely to be prescribed opioids, suggesting that gabapentinoids are not replacing opioid use, but merely adding to it. Alarmingly, 38% of patients prescribed gabapentinoids after a surgical procedure leading to a SNF stay had prolonged use (i.e., prescribing at > 90 days post-discharge). Further, though we did not control for prior opioid use, almost half (51.5%) of these patients also had prolonged opioid use—a potentially inappropriate and perhaps unintentional continuation of medications that have dangerous side effects in older adults.

Compared to other studies looking at postoperative prescribing of gabapentinoids for older adults discharging home, 4% is a larger proportion of prescribing. While the numbers are currently small, other studies have shown the use of gabapentin appears to be increasing over time [17], making this a potential over prescribing problem in the future. Additionally, the proportion of patients with prolonged use in this cohort is more than 1.5 times higher than that of another study looking at Medicare patients after surgery who were discharged from the hospital to home [18]. Again, while these numbers are small, the cumulative effects of the aging population, the increasing number of older adults undergoing surgery, and the increased need for post-acute SNF care in the postoperative period suggest prolonged use may further rise. This prolonged use may also be a signal that other medications are continued after SNF, leading to polypharmacy and adverse drug events. Patients with gabapentinoids were also more likely to be prescribed opioids, which is a concerning finding but perhaps not surprising given one study found that 70% of patients discharged from a SNF were prescribed an opioid [46].

The drivers of prolonged use (of both gabapentinoids and opioids) after SNF discharge are unclear but deserve further study. It's possible that medication reconciliation at admission to the SNF from the hospital is part of the problem, and certainly communication challenges between the hospital and SNF have been a consistent problem described in other studies [6, 12, 13]. Improving medication reconciliation in the SNF setting has been found to be challenging due to personnel and time constraints, complex workflows, and variation in the capabilities and communication between hospital and SNF electronic health records [47]. Other studies have identified poor or unidirectional communication or lack of agreement within discharge paperwork for medication discrepancies or errors [4, 6]. Importantly, there remain gaps in our understanding of medication management and workflow processes within SNFs highlighting a potential impactful area for future research.

Our study suggests there are opportunities to improve medication management for post-operative older adults as they transition from the hospital to SNF and transition from SNF to home. This data can only point out the prevalence of use and prolonged use, but the question of why this exists remains elusive. Though studies point to transitions of care being a time fraught with danger due to error, the specific process and use of pain medications in this transition remain unknown. It is possible that patients transitioning to SNF are sicker than those going home; however, we hypothesize that much of this prolonged use is unintentional and is due to miscommunication or misunderstanding of the need and use of gabapentin over these two care transitions. As non-opioid medications are increasingly used after surgery, and the population of older adults undergoing surgery grows, careful attention must be paid to ensuring that these medications are appropriately discontinued and not continued indefinitely.

## 5 ∣ Limitations

Our study has several limitations. We excluded patients with Medicare Advantage as diagnosis data from these outpatient visits are harder to obtain, which may limit our generalizability to fee-for-service beneficiaries. However, our sample size remains large despite this exclusion to allow robust assessments of the non-Medicare Advantage population. Finally, our ability to detect differences between racial/ethnic groups is limited by the manner in which Medicare traditionally categorized patients and has low validity in some groups [48]. Because of this, specific racial groups were too small of a subgroup to meaningfully interpret the results and were therefore removed from the analysis by race and ethnicity. We did not study preoperative or inpatient use of gabapentin. Of note, while our methods of assessing medication prescribing are based on established practice, it may not perfectly capture actual medication use as this dataset was not designed to do so.

## 6 ∣ Conclusions

In conclusion, in this national cohort of older adults undergoing a wide range of common surgical procedures who were discharged to SNF, we found that 4% received a new gabapentinoid prescription at SNF discharge, with 61% having concomitant opioid use. Of those with a new prescription, 38% had prolonged use, more than 1.5 times the proportion of prolonged use found in a different study among patients being discharged directly home. Importantly, patients who were sicker and more disadvantaged were more likely to have prolonged use. Our study highlights that additional work needs to focus on understanding and preventing inappropriate prolonged use of postoperative pain medications after SNF discharge.

## Supplementary Material

Supplementary Information

Additional supporting information can be found online in the Supporting Information section. **Table S1:** List of opioids used in analysis. **Table S2:** Included surgical procedures and groupings. **Table S3:** List of procedures using HCPCS/CPT codes, and inpatient procedures using ICD9-CM or ICD10-PCS codes. **Table S4:** Overall skilled nursing facility discharge cohort.

## Figures and Tables

**TABLE 1 ∣:** Cohort for older adults with prolonged use of gabapentinoids.

	Total	No prolonged use	Gabapentinoid prolonged use	
	*N* = 3182, col.%	1963 (61.7%), row%	1219 (38.3%), row%	*p*
Age, mean (sd)	76.0 (6.4)	76.0 (6.4)	76.0 (6.5)	0.9111
Sex				0.2314
Female	2441 (76.7%)	1492 (61.1%)	949 (38.9%)	
Male	741 (23.3%)	471 (63.6%)	270 (36.4%)	
Race				0.6841
White	2591 (81.4%)	1605 (61.9%)	986 (38.1%)	
Black	281 (8.8%)	171 (60.9%)	110 (39.1%)	
Hispanic	170 (5.3%)	107 (62.9%)	63 (37.1%)	
Other/unknown	140 (4.4%)	80 (57.1%)	60 (42.9%)	
Charlson Score				< 0.0001
0	611 (19.2%)	447 (73.2%)	164 (26.8%)	
1–2	1127 (35.4%)	734 (65.1%)	393 (34.9%)	
3–4	741 (23.3%)	422 (57%)	319 (43%)	
5+	703 (22.1%)	360 (51.2%)	343 (48.8%)	
Gabapentinoid days supply at discharge				
Median (q1, q3)	30 (20, 30)	30 (15, 30)	30 (30, 30)	< 0.0001
Concurrent opioid use				
Yes	1938 (60.9%)	1211 (62.5%)	727 (37.5%)	0.2488
Opioid days supply				
Median (q1, q3)	8 (5, 15)	7 (5, 14)	10 (6, 20)	< 0.0001
Opioid oral morphine equivalents (mg)				
Median (q1, q3)	300 (200, 600)	300 (200, 525)	375 (210, 675)	< 0.0001
Skilled nursing facility length of stay				
Median (q1, q3)	16 (11, 24)	15 (10, 21)	20 (13, 31)	< 0.0001
Surgery planned				< 0.0001
Yes	2814 (88.4%)	1808 (64.3%)	1006 (35.7%)	
Care complexity (number of physicians seen in prior 6 months)				< 0.0001
q1 (0–9)	767 (24.1%)	466 (60.8%)	301 (39.2%)	
q2 (10–13)	699 (22.0%)	477 (68.2%)	222 (31.8%)	
q3 (14–18)	789 (24.8%)	520 (65.9%)	269 (34.1%)	
q4 (19–119)	927 (29.1%)	500 (53.9%)	427 (46.1%)	
Type of procedure				< 0.0001
Orthopedic procedure	2726 (85.7%)	1773 (65.0%)	953 (35.0%)	
Spine procedure	363 (11.4%)	153 (42.1%)	210 (57.9%)	
Laparoscopic procedure	> 11	< 11	> 11	
Open abdominal procedure	54 (1.7%)	24 (44.4%)	30 (55.6%)	
Vascular	< 11	< 11	< 11	
Area deprivation index (patient zip code)				0.0001
Missing	25 (0.8%)	> 11	< 11	
1–20	627 (19.7%)	418 (66.7%)	209 (33.3%)	
21–40	827 (26.0%)	541 (65.4%)	286 (34.6%)	
41–60	855 (26.9%)	515 (60.2%)	340 (39.8%)	
61–80	698 (21.9%)	394 (56.4%)	304 (43.6%)	
81–100	150 (4.7%)	79 (52.7%)	71 (47.3%)	

Abbreviation: Ref, reference group.

**TABLE 2 ∣:** Multivariable model for prolonged use of gabapentinoids.

	Adjusted Odds Ratio	*p*
Age	0.99 (0.98,1.00)	0.1324
Sex		0.0321
Male	ref.	
Female	1.22 (1.02,1.47)	
Race		0.8017
White	ref.	
Non-white	1.03 (0.84,1.25)	
Gabapentinoid days supply		< 0.0001
1–7 days	ref.	
8–27 days	0.97 (0.69,1.37)	
28–45 days	1.37 (1.00,1.88)	
45+ days	5.22 (3.52,7.75)	
Opioid prescribing		0.7205
No	ref.	
Yes	1.03 (0.88,1.21)	
Surgery planned		< 0.0001
Yes	ref.	
No	2.02 (1.58,2.59)	
Type of procedure		< 0.0001
Orthopedic procedure	ref.	
Spine procedure	2.23 (1.75,2.84)	
Laparoscopic procedure	3.41 (1.45,8.03)	
Open abdominal procedure	1.88 (1.05,3.38)	
Vascular	1.29 (0.36,4.57)	
Area deprivation index (patient zip code)		0.0037
1–20	ref.	
21–40	0.98 (0.78,1.23)	
41–60	1.15 (0.91,1.44)	
61–80	1.32 (1.04,1.67)	

Abbreviation: Ref, reference group.

## Data Availability

This data is used through a DUA with Medicare and therefore is not our data to share. Should another research group want access to this data, they will need to contact Medicare directly. Siqi Gan and Dr. John Boscardin had full access to all the data in the study and take responsibility for the integrity of the data and the accuracy of the data analysis.

## Summary

- Key Points
  - Among patients discharged to SNF after a surgical procedure, 4% were prescribed gabapentinoids at discharge home.
  - Of those, 38% went on to have prolonged use (i.e., prescribing at > 90 days post-procedure).
  - Patients with gabapentinoids were also more likely to be prescribed opioids, suggesting that gabapentinoids are not replacing opioid use, but merely adding to it.
- Why Does This Matter?
  - Among older adults with a new gabapentinoid prescription discharging to SNF then home, 38% had prolonged use, more than 1.5 times the proportion of prolonged use found in a different study among patients being discharged directly home. As an increasing number of older adults undergo surgery and transition to SNF prior to home, the continuation of postoperative pain medications should be closely monitored to avoid adverse drug events or inadvertent continuation.
