# Supplementary material for: Prolonged Use of Postoperative Gabapentin After Discharge From a Skilled Nursing Facility
Source: J Am Geriatr Soc. Author manuscript; Available in PMC 2026 Jul 29. (PMC13417771; doi:10.1111/jgs.70578)
Supplement: Supplementary Information [file NIHMS2194996-supplement-Supplementary_Information.pdf]

eTable 1: List of opioids used in analysis

Butorphanol
Codeine
Dihydrocodeine
Fentanyl
Hydrocodone
Hydromorphone
Meperidine
Morphine
Oxycodone
Oxymorphone
Pentazocine
Tapentadol
Tramadol

eTable 2: Included Surgical Procedures and Groupings

Total Shoulder Arthroplasty	Orthopedic
Total Hip Arthroplasty	Orthopedic
Prostatectomy, Laparoscopic	Laparoscopic
Hysterectomy, Laparoscopic	Laparoscopic
Cholecystectomy, Laparoscopic	Laparoscopic
Initial Inguinal Hernia Repair, Open	Open Abdominal
Initial Inguinal Hernia Repair, Laparoscopic	Laparoscopic
Lumbar Laminotomy	Spine
Lumbar Laminectomy	Spine
Hysterectomy, Vaginal	Open Abdominal
Carotid Endarterectomy	Vascular
Total Knee Arthroplasty	Orthopedic
Low Anterior Resection, Laparoscopic	Laparoscopic
Ventral Hernia Repair, Open	Open Abdominal

eTable3: List of procedures using HCPCS/CPT codes, and inpatient procedures using ICD9-CM or ICD10-PCS codes

**Procedure**	**CPT code**	**ICD9/10**
Total Shoulder Arthroplasty	23472	81.51, 81.54, 81.88, 81.80, 81.81, 81.83, 81.23
Total Hip Arthroplasty	27130 , 27134	81.21, 81.4, 81.51
Prostatectomy, Laparoscopic	55866	60.2*, 60.3*, 60.4*, 60.5* 60.6*
Hysterectomy, Laparoscopic	58571	68.3*, 68.4*, 68.5*, 68.6*, 68.7*, 68.9*
Cholecystectomy, Laparoscopic	47562	51.23, 51.24
Initial Inguinal Hernia Repair, Open	49505	550*, 53.0*, 53.1*
Lumbar Laminotomy/Laminectomy	63030, 63047	03.0*, 03.4*, 03.5*, 03.6*
Hysterectomy, Vaginal	58262	
Carotid Endarterectomy	35301	38.02, 38.12, 38.32, 38.42, 38.62
Knee Arthroplasty	27447 , 27487	81.22, 81.43, 81.47, 81.54, 81.55
Low Anterior Resection, Laparoscopic	44207	48.0*, 48.1*, 48.4*, 48.5*, 48.6*
Initial Inguinal Hernia Repair, Laparoscopic	49650	17.1*, 17.2*
Ventral Hernia Repair, Open	49560	53.41, 53.49, 53.5*, 53.61, 53.69

eTable 4: Overall Skilled Nursing Facility Discharge Cohort

	Total<br>N=79417, col%	No gabapentinoid prescription<br>N=76235 (96.3%), row%	New gabapentinoid prescription<br>N=3182 (4.0%), row%	p-value
**Age, mean (sd)**	77.4 (6.8)	77.5 (6.8)	76.0 (6.4)	<0.0001
**Sex**				<0.0001
Female	58014 (73.0%)	55573 (95.8%)	2441 (4.2%)	
Male	21403 (27.0%)	20662 (96.5%)	741 (3.5%)	
**Race**				<0.0001
White	68390 (86.1%)	65799 (96.2%)	2591 (3.8%)	
Black	5090 (6.4%)	4809 (94.5%)	281 (5.5%)	
Hispanic	3341 (4.2%)	3171 (94.9%)	170 (5.1%)	
Other/Unknown*	2596 (3.3%)	2456 (94.6%)	140 (5.4%)	
**Charlson Score**				0.0032
0	16126 (20.3%)	15515 (96.2%)	611 (3.8%)	
1-2	29552 (37.2%)	28425 (96.2%)	1127 (3.8%)	
3-4	17959 (22.6%)	17218 (95.9%)	741 (4.1%)	
5+	15780 (19.9%)	15077 (95.5%)	703 (4.5%)	
**Gabapentinoid days supply**				
Median (q1, q3)	30 (20, 30)	N/A	30 (20, 30)	N/A
**Concurrent opioid use**				<0.0001
	33911 (42.7%)	31973 (94.3%)	1938 (5.7%)	
**Opioid days supply**				0.0023
Median (q1, q3)	8 (5, 15)	8 (5, 15)	8 (5, 15)	
**Opioid oral morphine equivalent (mg)**				0.0059
Median (q1, q3)	300 (210, 500)	300 (210, 500)	300 (200, 600)	
**Facility type**				0.1803
Inpatient	79191 (99.7%)	76022 (96%)	3169 (4%)	
Outpatient	226 (0.3%)	213 (94.2%)	13 (5.8%)	
**Skilled nursing facility length of stay**				
Median (q1, q3)	15 (10, 22)	15 (10, 22)	16 (11, 24)	<0.0001
**Surgery planned**				<0.0001
Yes	67830 (85.4%)	65016 (95.9%)	2814 (4.1%)	
No	11587 (14.6%)	11219 (96.8%)	368 (3.2%)	

**Care complexity (number of physicians seen in prior 6 months)**				<0.0001
q1 (0-9)	23637 (29.8%)	22870 (96.8%)	767 (3.2%)	
q2 (10-13)	19168 (24.1%)	18469 (96.4%)	699 (3.6%)	
q3 (14-18)	18710 (23.6%)	17921 (95.8%)	789 (4.2%)	
q4 (19-119)	17902 (22.5%)	16975 (94.8%)	927 (5.2%)	
**Type of procedure**				<0.0001
Orthopedic procedure	71437 (90.0%)	68711 (96.2%)	2726 (3.8%)	
Spine procedure	3132 (3.9%)	2769 (88.4%)	363 (11.6%)	
Laparoscopic procedure	1638 (2.1%)	1609 (98.2%)	29 (1.8%)	
Open abdominal procedure	2744 (3.5%)	2690 (98%)	54 (2%)	
Vascular	466 (0.6%)	456 (97.9%)	10 (2.1%)	
**Area deprivation index (patient zip code)**				<0.0001
Missing	658 (0.8%)	633 (96.2%)	25 (3.8%)	
1-20	12024 (15.1%)	11397 (94.8%)	627 (5.2%)	
21-40	19692 (24.8%)	18865 (95.8%)	827 (4.2%)	
41-60	23098 (29.1%)	22243 (96.3%)	855 (3.7%)	
61-80	20069 (25.3%)	19371 (96.5%)	698 (3.5%)	
81-100	3876 (4.9%)	3726 (96.1%)	150 (3.9%)	

*According to the Research Triangle Institute, this variable includes Asian/Pacific Islander, American Indian/Alaska Native, Unknown and 'Other.' Care complexity: (number of physicians seen in prior 6 months). sd = standard deviation. q = quartile. LOS = length of stay.

STROBE Statement—Checklist of items that should be included in reports of ***cohort studies***

	Item No	Recommendation	Page No
**Title and abstract**	1	(*a*) Indicate the study's design with a commonly used term in the title or the abstract<br>(*b*) Provide in the abstract an informative and balanced summary of what was done and what was found	2
**Introduction**			
Background/rationale	2	Explain the scientific background and rationale for the investigation being reported	4
Objectives	3	State specific objectives, including any prespecified hypotheses	5
**Methods**			
Study design	4	Present key elements of study design early in the paper	5
Setting	5	Describe the setting, locations, and relevant dates, including periods of recruitment, exposure, follow-up, and data collection	5-6
Participants	6	(*a*) Give the eligibility criteria, and the sources and methods of selection of participants. Describe methods of follow-up<br>(*b*) For matched studies, give matching criteria and number of exposed and unexposed	6
Variables	7	Clearly define all outcomes, exposures, predictors, potential confounders, and effect modifiers. Give diagnostic criteria, if applicable	7-8
Data sources/ measurement	8*	For each variable of interest, give sources of data and details of methods of assessment (measurement). Describe comparability of assessment methods if there is more than one group	8
Bias	9	Describe any efforts to address potential sources of bias	8
Study size	10	Explain how the study size was arrived at	6-8
Quantitative variables	11	Explain how quantitative variables were handled in the analyses. If applicable, describe which groupings were chosen and why	8
Statistical methods	12	(*a*) Describe all statistical methods, including those used to control for confounding<br>(*b*) Describe any methods used to examine subgroups and interactions<br>(*c*) Explain how missing data were addressed<br>(*d*) If applicable, explain how loss to follow-up was addressed<br>(*e*) Describe any sensitivity analyses	8
**Results**			
Participants	13*	(a) Report numbers of individuals at each stage of study—eg numbers potentially eligible, examined for eligibility, confirmed eligible, included in the study, completing follow-up, and analysed<br>(b) Give reasons for non-participation at each stage<br>(c) Consider use of a flow diagram	9
Descriptive data	14*	(a) Give characteristics of study participants (eg demographic, clinical, social) and information on exposures and potential confounders<br>(b) Indicate number of participants with missing data for each variable of interest<br>(c) Summarise follow-up time (eg, average and total amount)	9
Outcome data	15*	Report numbers of outcome events or summary measures over time	9-10

Main results	16	(*a*) Give unadjusted estimates and, if applicable, confounder-adjusted estimates and their precision (eg, 95% confidence interval). Make clear which confounders were adjusted for and why they were included<br>(*b*) Report category boundaries when continuous variables were categorized<br>(*c*) If relevant, consider translating estimates of relative risk into absolute risk for a meaningful time period	9-10
Other analyses	17	Report other analyses done—eg analyses of subgroups and interactions, and sensitivity analyses	9-10
**Discussion**			
Key results	18	Summarise key results with reference to study objectives	11
Limitations	19	Discuss limitations of the study, taking into account sources of potential bias or imprecision. Discuss both direction and magnitude of any potential bias	13
Interpretation	20	Give a cautious overall interpretation of results considering objectives, limitations, multiplicity of analyses, results from similar studies, and other relevant evidence	11-12
Generalisability	21	Discuss the generalisability (external validity) of the study results	13
**Other information**			
Funding	22	Give the source of funding and the role of the funders for the present study and, if applicable, for the original study on which the present article is based	15

*Give information separately for exposed and unexposed groups.

Note: An Explanation and Elaboration article discusses each checklist item and gives methodological background and published examples of transparent reporting. The STROBE checklist is best used in conjunction with this article (freely available on the Web sites of PLoS Medicine at http://www.plosmedicine.org/, Annals of Internal Medicine at http://www.annals.org/, and Epidemiology at http://www.epidem.com/). Information on the STROBE Initiative is available at http://www.strobe-statement.org.
